# Reduced miR-371b-5p expression drives tumor progression via CSDE1/RAC1 regulation in triple-negative breast cancer

**DOI:** 10.1038/s41388-022-02326-6

**Published:** 2022-04-30

**Authors:** Yesol Kim, Je Yeong Ko, Soo-Been Lee, Sumin Oh, Jee Won Park, Hyeok-Gu Kang, Da-Hyun Kim, Daeun Chung, Sera Lim, Hyunkyung Kong, Jongmin Kim, Kyung Hyun Yoo, Wonshik Han, Kyung-Hee Chun, Jong Hoon Park

**Affiliations:** 1 Department of Biological Science, Sookmyung Women’s University, Seoul, Republic of Korea; 2 Department of Biochemistry and Molecular Biology, Graduate School of Medical Science, Brain Korea 21 Project, Yonsei University College of Medicine, Seoul, Republic of Korea; 3 Cancer Research Institute, Seoul National University College of Medicine, Seoul, Republic of Korea; 4 Department of Surgery, Seoul National University College of Medicine, Seoul, Republic of Korea; 5 Research Institute of Women’s Health, Sookmyung Women’s University, Seoul, Republic of Korea

**Keywords:** Prognostic markers, Breast cancer

## Abstract

Triple-negative breast cancer (TNBC) is the most aggressive subtype of breast cancer; however, specific prognostic biomarkers have not yet been developed. In this study, we identified dysregulated microRNAs (miRNAs) in TNBC by profiling miRNA and mRNA expression. In patients with TNBC, miR-371b-5p expression was reduced, and miR-371b-5p overexpression significantly mitigated TNBC cell growth, migration, and invasion. In addition, we found that expression of cold shock domain-containing protein E1 (CSDE1), a direct target gene of miR-371b-5p, was upregulated in TNBC cells, and inhibition of CSDE1 expression alleviated TNBC cell growth by regulating *RAC1* transcription. Mechanistically, CSDE1, phosphorylated C-terminal domain (p-CTD) of RNA polymerase II (RNAPII), and CDK7 form a complex, and downregulation of CSDE1 leads to weak interaction between RNAPII p-CTD and CDK7, resulting in a decrease in RNAPII p-CTD expression to reduce *RAC1* transcript levels in CSDE1-deficient TNBC cells. Our data demonstrate that miR-371b-5p is a tumor-suppressive miRNA that regulates the CSDE1/Rac1 axis and could be a potential prognostic biomarker for TNBC.

## Introduction

Triple-negative breast cancer (TNBC) is defined as a tumor that lacks estrogen receptor, progesterone receptor, and human epidermal growth factor receptor (HER2) expression and represents approximately 15%–20% of breast cancer cases [1, 2]. Currently, no targeted therapy is available for patients with TNBC. Chemotherapy is the mainstay of treatment for TNBC. However, substantial number of patients with TNBC develop recurrence after adjuvant therapy [3]. Moreover, chemotherapy leads to worse outcomes in these patients than other subtypes because TNBC is a heterogeneous tumor classified into various subgroups [4, 5]. These challenges highlight the importance of research to identify an effective therapeutic strategy and elucidate the mechanism underlying TNBC aggressiveness.

MicroRNAs (miRNAs) are 20–22 nucleotide long noncoding RNAs. Most miRNAs silence their respective genes by annealing in the 3’-untranslated region (UTR) of protein-coding mRNAs [6]. In cancer, many miRNAs are dysregulated and regulate the expression of oncogenes or tumor-suppressor genes [6, 7]. Several dysregulated miRNAs are known to be involved in metastasis, invasion, and chemoresistance in breast cancer [8–10]. In addition, several global profiling of miRNA have identified the role of oncogenic and tumor-suppressive miRNAs in breast cancer, depending on molecular subtype, hormone receptor status, tumor stage, and resistance to chemotherapy to identify prognostic markers, as well as understanding the mechanisms related to dysregulated miRNAs [11, 12].

Cold shock domain-containing protein E1 (CSDE1), known as upstream of N-Ras (UNR), is a conserved RNA-binding protein (RBP) involved in several steps of post-translational regulation. Many RBPs bind to RNA secondary structures or sequence-specific motifs through individual RNA-binding domains [13, 14]. CSDE1 contains five cold shock domains that bind to single-stranded RNA [15] to modulate translation initiation, and alters the stability or abundance of target genes [16]. Several studies have revealed that abnormal expression of RBPs is associated with cancer prognosis [17, 18]. Likewise, CSDE1 acts as a proto-oncogene by regulating c-myc, c-fos, RAC1, and vimentin expression and promotes metastasis and invasion in cancer. In mammals, CSDE1 modulates cell apoptosis and differentiation depending on the cell type [19–21].

Here, we discovered a reduction in miR-371b-5p expression through microRNA profiling in tissue samples from patients with TNBC. Integrative analysis of miRNA and mRNA microarray revealed that CSDE1 is a direct target of miR-371b-5p, and its expression is highly upregulated in TNBC. Interestingly, we found that CSDE1 at upregulated levels could act as an oncogene to regulate tumor cell growth, migration, and invasion by modulating the transcription of *RAC1*, a well-known proto-oncogene. In addition, we revealed that downregulation of CSDE1 expression reduced phosphorylation of the C-terminal domain (CTD) of RNA polymerase II (RNAPII) by decreasing the interaction between phosphorylated (p)-CTD of RNAPII and cyclin-dependent kinase 7 (CDK7), resulting in downregulation of the *RAC1* transcript in CSDE1-deficient TNBC cells. Collectively, we showed that dysregulated miR-371b-5p/CSDE1/RAC1 axis deepens tumor progression and aggressiveness in patients with TNBC and suggested that miR-371b-5p is a potential prognostic biomarker for TNBC.

## Results

### Reduced expression of miR-371b-5p is associated with poor clinical outcomes in TNBC

**Fig. 1:**
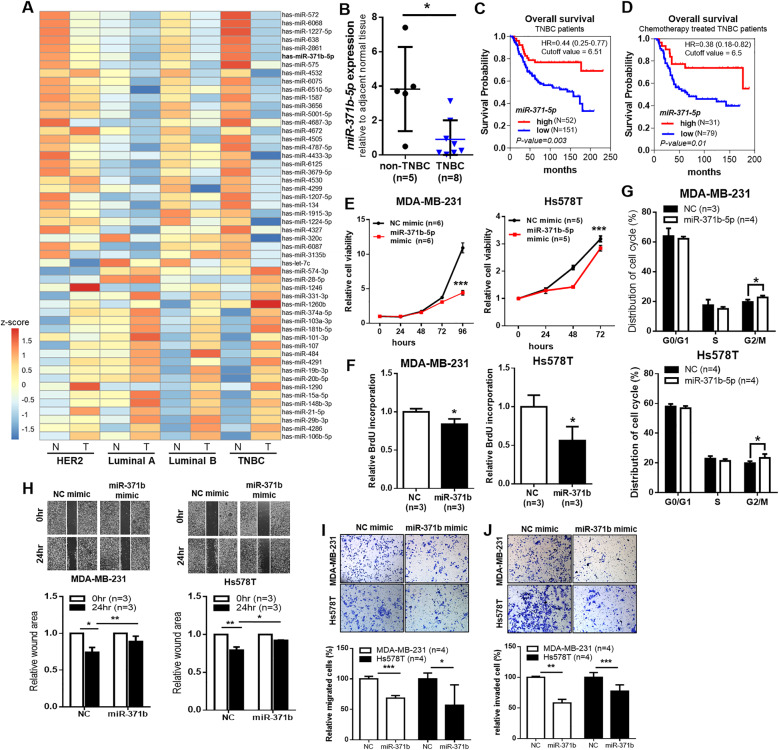
miR-371b-5p expression is downregulated and related to poor prognosis in TNBC. **A** A heatmap showing the mean values of 52 miRNAs in matched normal tissues and tumors in four breast cancer subtypes (Luminal A; Luminal B; HER2, epidermal growth factor 2-enriched cancer; and TNBC, triple-negative breast cancer). Z-score (normalized expression levels) are represented by colored bars; higher (red) or lower (blue). **B** TaqMan quantitative RT-PCR analysis of miR-371b-5p in non-TNBC and TNBC tissues. Data are presented as mean ± SD. **C** and **D** Kaplan–Meier analysis of overall survival of patients with TNBC and patients who underwent chemotherapy. *p* value was calculated using the log-rank test. Patients were stratified into “low” (blue) and “high” (red) miR-371-5p expression groups based on autoselect best cutoff. **E** CCK-8 assay to measure the cell viability in MDA-MB-231 and Hs578T cells after treatment with miR-371b mimics or negative control (NC) mimic. Data are presented as mean ± SD with 5 or 6 biological replicates. **F** Relative BrdU incorporation in MDA-MB-231 and Hs578T after treatment with miR-371b mimics or negative control. Data are presented as mean ± SD with 3 biological replicates. **G** The percentage of cells in each cell cycle phase was analyzed by flow cytometry. Data are presented as mean ± SD with 3 or 4 biological replicates. **H** Wound healing assay of a confluent culture of MDA-MB-231 and Hs578T cells with ectopic expression of miR-371b-5p at 0 and 24 h after scratching. The bar graph indicated the relative wound healing area. Data are presented as mean ± SD with 3 biological replicates. **I** Migrated cells with miR-371b mimic or negative control was measured using Transwell migration assay. The bar graph showed the proportion of relative migrated cells (%). **J** The proportion of invaded cells calculated based on overexpression of miR-371b-p or negative control is depicted in the bar graph. **I**, **J** The quantification data are presented as mean ± SD with 4 biological replicates. *p < 0.05, **p < 0.01, ***p < 0.005. Images are representative of each experiment.

To identify miRNAs that might be aberrantly expressed in TNBC, we profiled miRNA expression using miRNA microarray in four different breast cancer tumors along with the adjacent normal tissues (luminal A, luminal B, HER2, and TNBC). We used 10 tumors and their paired adjacent normal tissues for miRNA microarray analysis. The number and types of patients used in this analysis was as follows: luminal A (*n* = 2), luminal B (*n* = 2), HER2 (*n* = 3), TNBC (*n* = 3). Fifty-two miRNAs (|FC| > 2, *p* value < 0.05) were identified in TNBC tumor tissues; 21 miRNAs were upregulated and 31 miRNAs were downregulated more than twofold in the TNBC tumors compared with the matched normal tissues (Fig. 1A, Supplementary Fig. 1A). Among these 52 miRNAs, 27 miRNAs, including 9 upregulated miRNAs and 18 downregulated miRNAs, were found to be differentially expressed (|FC| > 2) in the TNBC subtype compared to other subtypes (Supplementary Fig. 1B). Of these miRNAs, we selected miR-371b-5p that was downregulated in TNBC.

To confirm reduced expression of miR-371b-5p in TNBC clinical cases, we observed its expression in TNBC tissues. The expression of miR-371b-5p in the adjacent normal tissues was compared with that in tumors in clinical tissue specimens (non-TNBC [luminal and HER2 subtype] vs. TNBC) and observed to be significantly downregulated in TNBC than in other breast cancer subtypes (Fig. 1B). Next, in order to explore the role of miR-371b-5p in clinical outcomes, we performed KM meta-analysis using the KM plot database [22]; the results revealed that low expression of miR-371-5p, which is the pre-miRNA of miR-371b-5p, was associated with poor OS in patients with TNBC (Fig. 1C). Furthermore, patients with low expression of miR-371-5p showed poor prognosis during chemotherapy (Fig. 1D). These findings suggest a potential role of miR-371b-5p in tumor suppression during TNBC progression.

To determine the pathological significance of miR-371b-5p in TNBC, we assessed tumor growth properties, including cell proliferation, migration, and invasive tumor growth, through overexpression of miR-371b-5p in TNBC cells. Cancer cell viability was significantly reduced in the miR-371b-5p overexpression group in MDA-MB-231 and Hs578T cells (Fig. 1E). Apoptosis and cell proliferation induce changes in cancer cell viability. Here, miR-371b-5p reduced cell proliferation, but not apoptosis, in MDA-MB-231 and Hs578T cells (Fig. 1F; Supplementary Fig. 2). Analysis of the cell cycle using flow cytometry showed an increase in the G2/M phase of cells upon ectopic expression of miR-371b-5p (Fig. 1G). Moreover, results of the wound healing assay demonstrated that ectopic expression of miR-371b-5p significantly decreased the migration of MDA-MB-231 and Hs578T cells (Fig. 1H). The number of migrated and invaded TNBC cells was considerably reduced after treatment with miR-371b-5p mimics, as examined by the Transwell assay (Fig. 1I, J). These results supported tumor-suppressive effect of miR-371b-5p in TNBC in vitro.

### Increased miR-371b-5p expression mitigates aggressive growth of TNBC in vivo

**Fig. 2:**
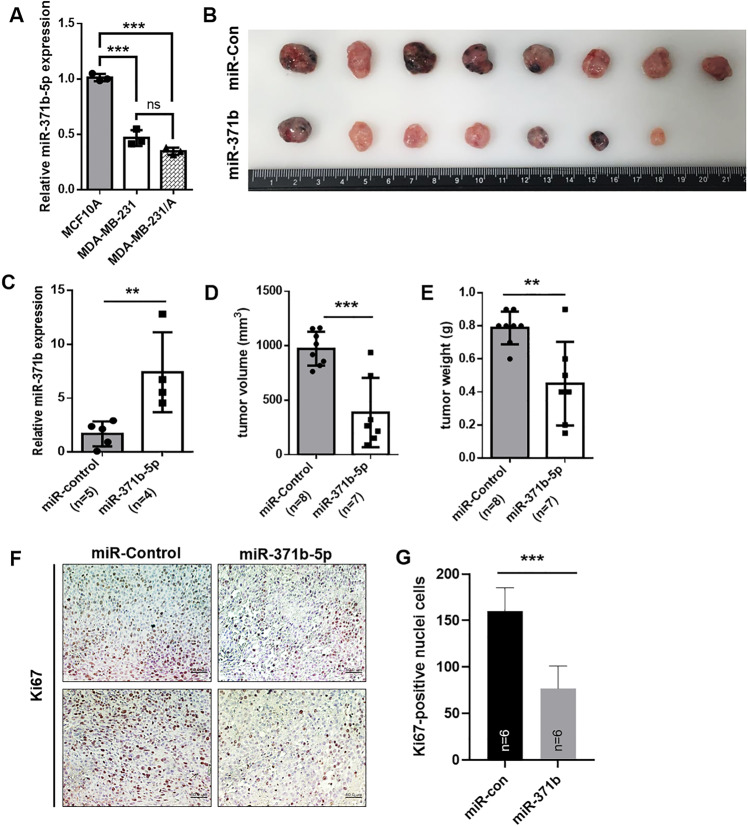
Increased miR-371b-5p expression inhibits TNBC growth in vivo. **A** Relative expression of miR-371b-5p in MCF10A, MDA-MB-231, and MDA-MB-231/A cells. Data are presented as mean ± SD with 3 technical replicates. **B** The images of tumors from xenograft mice (miR-control and miR-371b-5p). Xenograft experiment has been conducted with MDA-MB-231/Adaptation cells (MDA-MB-231/A) **C** Relative miR-371b-5p expression in control tumors and tumors with stably upregulated miR-371b-5p expression in xenograft mice. **D** and **E** Comparison of tumor volume and weight between miR-control group and miR-371b-5p group. **F** Representative immunohistochemistry (IHC) images of Ki67 staining in paraffin-embedded sections from xenograft mice. **G** The bar graph illustrates Ki67-positive cells in each xenograft group. Scale bar: 100 μm. **C**–**E**, **G** Data are presented as mean ± SD. *p < 0.05, **p < 0.01, ***p < 0.005. Images are representative of each experiment.

To assess miR-371b-5p function in vivo, stable cells expressing miR-371b-5p were cultured in MDA-MB-231/Adaptation cells (MDA-MB-231/A), derived from mouse-adapted MDA-MB-231 cells, which can be more aggressive than the primary MDA-MB-231 cells [23]. The results showed that miR-371b-5p expression in MDA-MB-231/A cells was reduced compared to that in MCF10A cells (non-malignant breast epithelial cells), but was maintained at a level similar to that in MDA-MB-231 cells (Fig. 2A). Two months after the injection, the primary tumor growth was significantly reduced in miR-371b-5p-overexpressed xenograft mice (Fig. 2B). We confirmed that miR-371b-5p was highly overexpressed in isolated miR-371b tumors compared to that in miR-con tumors (Fig. 2C). Both tumor volume and weight decreased in the miR-371b-5p overexpression group (Fig. 2D, E). In addition, staining with the cancer cell proliferation marker *Ki67* revealed that the proliferative capacity was impaired in xenograft mice with upregulated miR-371b-5p expression (Fig. 2F, G). Collectively, the gain-of-function in vivo study demonstrated that lower miR-371b-5p expression prompted cancer cell growth in TNBC.

### CSDE1, direct target of miR-371b-5p, is overexpressed in TNBC

**Fig. 3:**
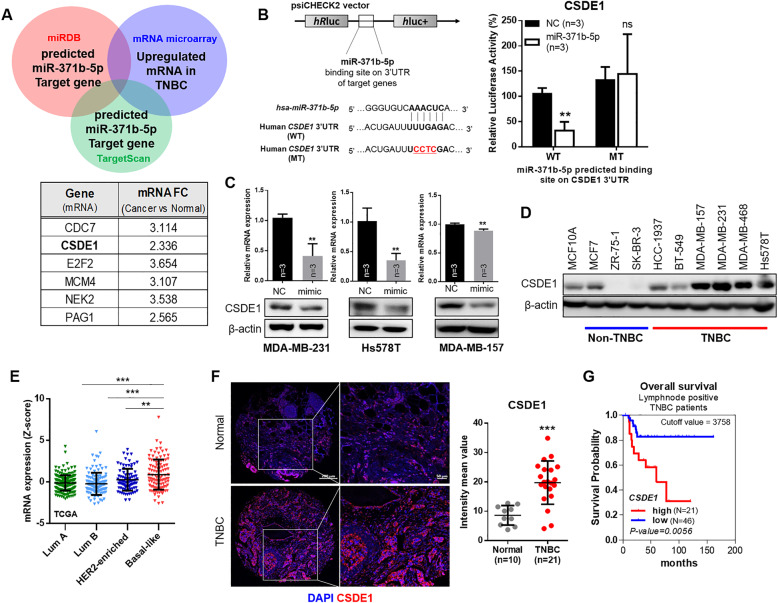
miR-371b-5p directly regulates CSDE1 in TNBC cells. **A** The Venn diagram showed the approach of miR-371b-5 target gene selection. Gene list and mRNA expression change data from mRNA microarray. **B** The sequence alignment of miR-371b-5p and 3′UTR of human CSDE1 including miR-371b-5-binding site. Luciferase activity after transfecting HEK293T cells with miR-371b-5p mimics and wild-type (WT) or mutant (MT) constructs in 48 h. Data are presented as mean ± SD with 3 biological replicates. **C** The expression of CSDE1 mRNA and protein after transfection of MDA-MB-231, Hs578T, and MDA-MB-157 cells with miR-371b-5p mimic. Data are presented as mean ± SD with 3 biological replicates. **D** The expression of CSDE1 in non-TNBC and TNBC cell lines. **E** TCGA data analysis showing the CSDE1 expression level. Data are presented as mean ± SD in breast cancer subtypes from TCGA dataset. Lum A, luminal A; Lum B, luminal B; HER2-enriched, HER2-enriched subtype; Basal-like, basal-like subtype. **F** Immunofluorescence staining of CSDE1 in tissue microarray (TMA) samples (left). The graph indicates the mean intensity of CSDE1 fluorescence in normal and TNBC tissues (right). Data are presented as mean ± SD. **G** Kaplan–Meier analysis of the overall survival depending on CSDE1 expression in lymph node-positive patients with TNBC. *p < 0.05, **p < 0.01, ****p < 0.0001; ns, not significant.

To investigate miR-371b-5p targets, mRNA microarray was performed and analyzed by integrating the miRNA profiling data. The list of upregulated mRNAs in TNBC was sorted through comparison between tumors of patients with TNBC and matched normal tissues (*p* < 0.05, fold change > 1.5). The list of overexpressed genes in TNBC tumors was compared with miRDB (Ver. 3.0) data for identifying a druggable target of miR-371b-5p in the selected gene set; we then narrowed down and selected the potential target genes of miR-371b-5p—*CSDE1*, *CDC7*, *NEK2*, *PAG1*, *E2F2*, and *MCM4*—using the TargetScan data (Fig. 3A). To identify the specific target genes, the predicted miR-371b-5p-binding site on the 3′UTR of each gene was cloned for luciferase reporter assay. The results showed that the ectopic miR-371b-5p significantly repressed the luciferase activity in the 3′UTR of the *CSDE1* wild-type (WT) construct containing miR-371b-5p-binding site (Fig. 3B). Contrarily, the luciferase activity was not inhibited in constructs of *MCM4*, *CDC7*, *E2F2*, *NEK2*, and *PAG1* 3′UTR (Supplementary Fig. 3a). Moreover, *CSDE1* expression was markedly reduced by the ectopic expression of miR-371b-5p in TNBC cells (Fig. 3C). However, *MCM4*, *CDC7*, *E2F2*, *NEK2* and *PAG1* expression did not show a pattern that was commonly reduced in miR-371b-5p-overexpressed TNBC cells (Supplementary Fig. 3b). These results suggest that *CSDE1* is a direct target of miR-371b-5p in TNBC. In addition, *CSDE1* expression was upregulated in TNBC cell lines compared to that in non-TNBC cell lines and the non-cancerous breast cancer MCF10A cells (Fig. 3D). Expanded analyses using the TCGA dataset [24] further supported that *CSDE1* was overexpressed in the basal-like subtype compared to that in other subtypes (luminal A, luminal B, and HER2) in clinical samples (Fig. 3E). Furthermore, we used tissue microarray slides to analyze CSDE1 expression in TNBC tissues, and found that CSDE1 staining intensity was significantly higher in TNBC tissues than in adjacent normal tissues (Fig. 3F). In addition, Kaplan–Meier analysis of the overall survival of patients with high CSDE1 expression showed poor prognosis with lymph node metastasis (Fig. 3G). These prognostic values indicated that high expression of CSDE1 was strongly associated with tumor progression and might be related to metastatic probability in TNBC. These results suggest that *CSDE1* is the target gene of miR-371b-5p with aberrantly reduced expression and could affect cells to promote TNBC progression.

### Knockdown of *CSDE1* induces a decrease in cell proliferation, migration, and invasion

**Fig. 4:**
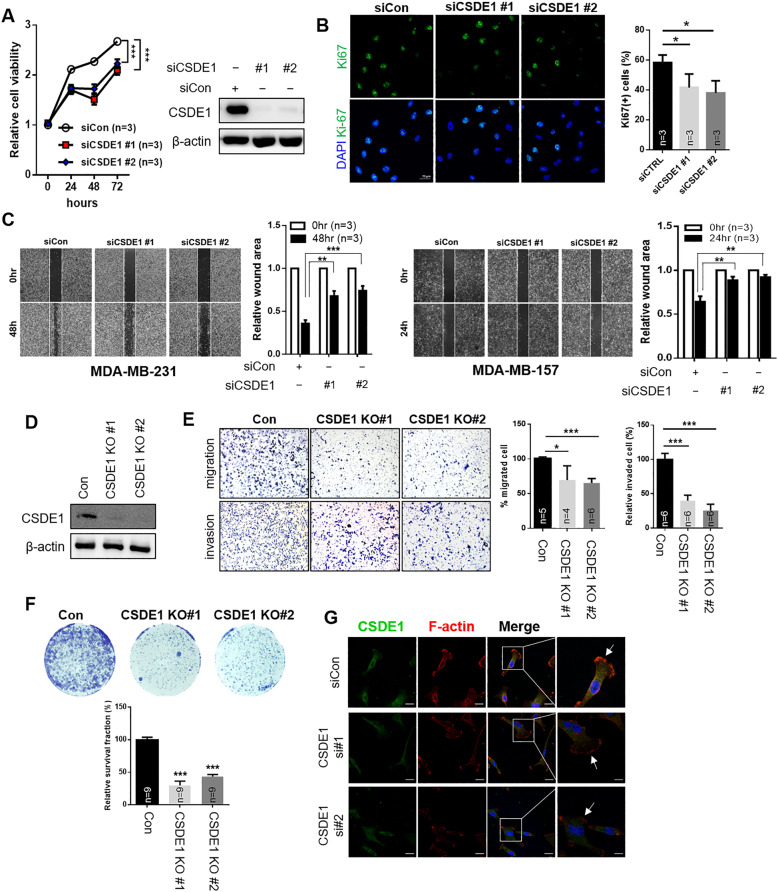
Inhibition of CSDE1 expression alleviates cancer progression. **A** The cell viability was measured by inhibiting CSDE1 expression using two independent siRNAs in MDA-MB-231 cells (left). Data are presented as mean ± SD with 3 biological replicates. The expression of CSDE1 was measured after siRNA treatment by western blotting (right). **B** Representative images of Ki67 staining of MDA-MB-231 cells treated with two independent *CSDE1* siRNAs (left). The bar graph indicates the percentage of Ki67-positive cells in each group (right). Data are presented as mean ± SD with 3 biological replicates. **C** Wound healing assay of MDA-MB-231 and MDA-MB-157 cells treated with two *CSDE1* siRNAs after scratching the confluent cells. After indicated time points, the wound closure area was measured using Image J. Data are presented as mean ± SD with 3 biological replicates. **D** CSDE1 expression in each stable *CSDE1*-knockout and control MDA-MB-231 cell (con, control; CSDE1 KO#1, CSDE1-knockout #1; and CSDE1 KO#2, CSDE1-knockout #2). **E** The representative image of migrated and invaded cells in stable *CSDE1*-knockout and control cells (left). Quantification of cells through migration and invasion assays was performed (right). Data are presented as mean ± SD with 4 to 6 biological replicates. **F** Representative images indicate colony formation in each cell. The bar graph shows relative survival fraction in each group. Data are presented as mean ± SD with 9 biological replicates. **G** Immunofluorescence staining of F-actin and CSDE1 after siRNA treatment. Enlarged image of each group is shown in the rightmost panel. The pointed arrows indicate lamellipodium staining (F-actin). *p < 0.05, **p < 0.01, ***p < 0.005.

Based on the analysis of the prognostic value of CSDE1, we suggested that high expression of CSDE1 could affect cancer cell progression in TNBC. Therefore, we attempted to observe the biological function of CSDE1 by attenuating its expression in TNBC cells. First, cancer cell viability and Ki67-positive cells were significantly impaired in MDA-MB-231 cells treated with *CSDE1* siRNAs (Fig. 4A, B). As high *CSDE1* expression was associated with poor prognosis in lymph node-positive patients, we investigated the contribution of CSDE1 in cancer cell migration and metastasis in TNBC. The wound healing assay indicated that *CSDE1*-knockdown cells inhibited the migration of mesenchymal-like TNBC cells, MDA-MB-231 and MDA-MB-157 (Fig. 4C). We generated the knockout MDA-MB-231 cells targeting *CSDE1* for further investigation and confirmed that the expression of *CSDE1* was attenuated in these cell lines (Fig. 4D). The migration and invasion assays supported that the knockout of *CSDE1* led to a reduction in the migration and invasion ability of TNBC cells (Fig. 4E). Furthermore, the clonogenic assay revealed that knockout of *CSDE1* prominently reduced the colony-forming ability of TNBC cells (Fig. 4F).

In cancer cells, actin cytoskeleton components undergo changes to survive and migrate for metastasis [25]. Immunostaining of F-actin revealed change in lamellipodium, an actin cytoskeletal protein in the leading cell edge that contributes to cell migration and metastasis in cancer [26]. In cells with *CSDE1* knockdown, the intensity of F-actin staining was significantly reduced in the leading cell edge (Fig. 4G). These data suggested that CSDE1 promoted cancer cell migration and metastasis through remodeling of actin cytoskeleton, particularly in lamellipodia. Collectively, these findings suggest that CSDE1 exhibits oncogenic activity by regulating invasive capacity and is required to promote aggressive growth of TNBC cells.

### CSDE1 binds to RAC1 to regulate TNBC progression in vitro

**Fig. 5:**
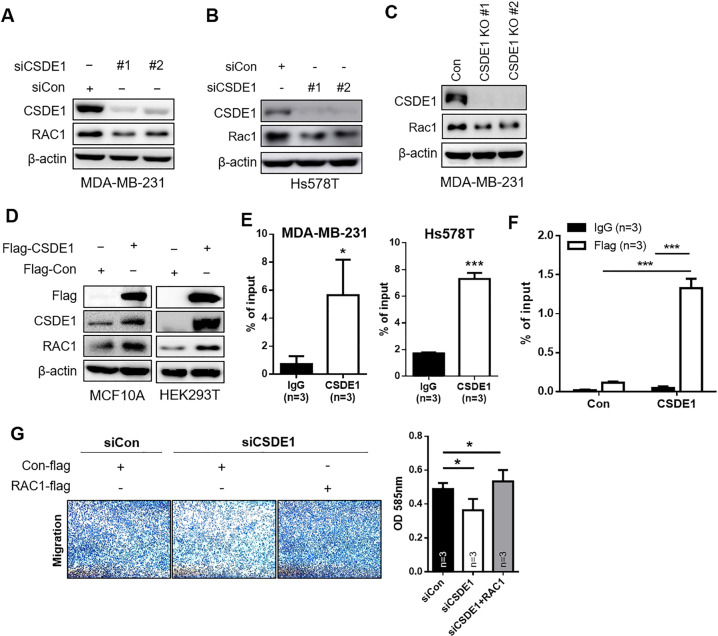
CSDE1 induced TNBC progression via Rac1 regulation. **A** and **B** The expression of CSDE1 and Rac1 was analyzed via western blotting after knockdown of *CSDE1* expression using two independent siRNAs in MDA-MB-231 and Hs578T cells. **C** Western blot analysis of CSDE1 and Rac1 by inhibiting *CSDE1* expression using lentivirus Cas9/CRISPR targeting of CSDE1. β-actin was used as loading control. **D** The expression of CSDE1 and Rac1 in MCF10A and HEK293T cells using Flag-tagged CSDE1 vector. **E** qPCR of *Rac1* mRNA enrichment (% of input) after RNA pull-down by IgG and CSDE1 antibody in MDA-MB-231 and Hs578T cells. Data are presented as mean ± SD with 3 biological replicates. **F** The enrichment of *Rac1* mRNA was measured via qPCR after RNA pull-down by IgG and Flag antibody in cells overexpressing of Flag-tagged CSDE1 or control (Con) vector. Data are presented as mean ± SD with 3 biological replicates. A 10% input was used for measurement in **E** and **F**. **G** Transwell assay for analyzing migration ability in the indicated groups of MDA-MB-231 cells (left). Quantitative analysis of migrated cells using microplate reading (right). Data are presented as mean ± SD with 3 biological replicates. *p < 0.05, ***p < 0.005.

Previous studies have shown that upregulated CSDE1 expression was associated with melanoma through elongation of the oncogene Rac1 [20]. Rac1 has a crucial role in regulating actin structures, including lamellipodia [27]. To explore the mechanism underlying CSDE1-related tumor progression in TNBC, we verified that Rac1 expression was regulated by CSDE1 in TNBC. We observed that Rac1 expression was significantly decreased in *CSDE1*-knockdown MDA-MB-231 and Hs578T cells (Fig. 5A, B) and in *CSDE1*-knockout cell lines (Fig. 5C). In addition, we found that Rac1 expression was elevated in *CSDE1*-overexpressed MCF10A (non-malignant breast epithelial cells) and HEK293T cells (Fig. 5D). These results indicate that CSDE1 is involved in regulating Rac1 expression.

Next, we investigated whether CSDE1 is involved in regulation of Rac1 expression through direct binding to *Rac1* mRNA. Through the RIP assay, endogenous CSDE1 was found to directly bind to *Rac1* mRNA transcripts in two TNBC cell lines (Fig. 5E). The enrichment of *Rac1* mRNA was high in the cells expressing exogeneous Flag-tagged *CSDE1* (Fig. 5F). To elucidate whether CSDE1 regulates cancer malignancy by regulating Rac1 expression, we rescued *Rac1* expression in *CSDE1*-knockdown cells. *Rac1*-overexpressing *CSDE1*-knockdown cells showed increased migration ability compared to *CSDE1*-knockdown cells (Fig. 5G), indicating that CSDE1 promotes TNBC migration via Rac1. Thus, we suggest that CSDE1 expressed at high levels directly binds to *Rac1* mRNA and induces its expression to promote the aggressiveness of TNBC cells.

### CSDE1 meditates *Rac1* transcription by binding to p-RNAPII

**Fig. 6:**
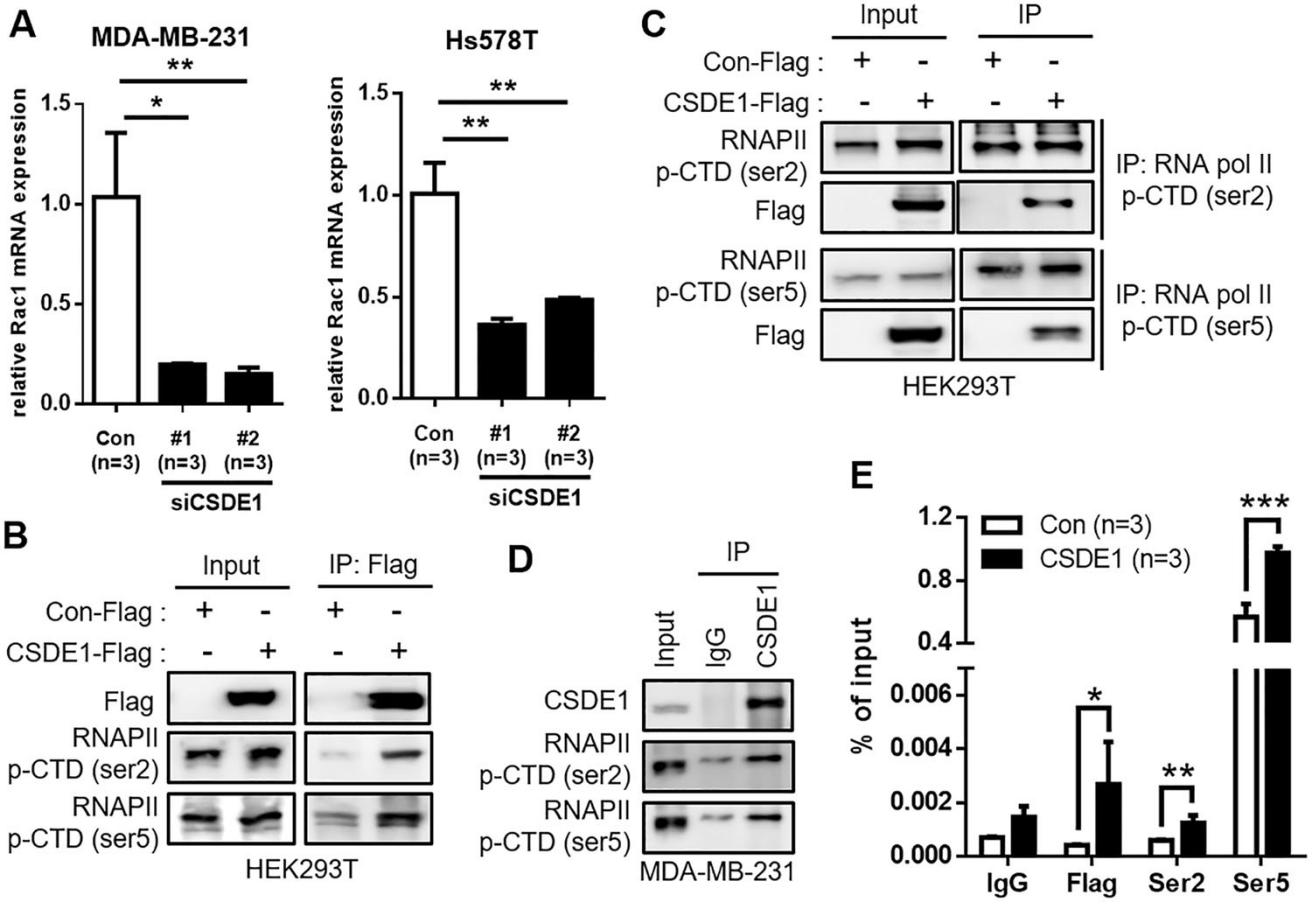
CSDE1 interacts with RNAPII and *Rac1* mRNA. **A** Relative mRNA expression of Rac1 in *CSDE1* siRNA-treated MDA-MB-231 and Hs578T cells. Data are presented as mean ± SD with 3 biological replicates. **B**, **C** Interaction of CSDE1 Flag with RNAPII p-CTD (ser2, ser5) in HEK293T cells transfected with control or CSDE1-Flag-tagged vector. **D** Interaction of endogenous CSDE1 with RNAPII p-CTD (ser2, ser5) in MDA-MB-231 cells. **E** The enrichment of *Rac1* mRNA was measured via qPCR after RNA pull-down by IgG, Flag, RNAPII p-CTD (ser2), and RNAPII p-CTD (ser5) antibodies in HEK293T cells transfected with control or CSDE1-Flag-tagged vector. A 10% input was used for measurement in **E**. Data are presented as mean ± SD with 3 technical replicates. *p < 0.05, ***p < 0.005.

Reportedly, CSDE1 acts as a connector for RNA and protein regulators [28]. To decipher the effect of CSDE1 on Rac1 expression, we first examined whether CSDE1 could regulate Rac1 at the translational level. Although ubiquitination and biosynthesis were blocked after treatment with MG132 and cycloheximide, respectively, Rac1 expression was consistently downregulated in *CSDE1*-knockdown MDA-MB-231 cells (Supplementary Fig. 4a, b). We confirmed that *Rac1* mRNA stability did not change by inhibiting mRNA synthesis in *CSDE1* siRNA-transfected cells (Supplementary Fig. 4c). Furthermore, *Rac1* mRNA expression was decreased in *CSDE1*-knockdown TNBC cells (Fig. 6A). Based on these data, we hypothesized that CSDE1 directly regulates Rac1 expression by acting as a bridge that connects transcription-related factors.

RNAPII p-CTD plays essential role in the regulation of gene transcription [29]. However, it is unclear whether CSDE1 binds to RNAPII p-CTD. We found that CSDE1 was bound to the p-CTD of RNAPII through immunoprecipitation (Fig. 6B). Conversely, pull-down assays with each p-RNAPII showed binding with CSDE1 (Fig. 6C). Moreover, we observed that endogenous CSDE1 directly interacted with RNAPII p-CTD in MDA-MB-231 cells (Fig. 6D). Next, we evaluated each RNAPII p-CTD complex bound to Rac1 transcripts. Each RNAPII p-CTD was highly enriched with *Rac1* mRNA, as determined by RIP-qPCR (Fig. 6E). Collectively, CSDE1 regulated Rac1 expression by tightly binding to the *Rac1* mRNA transcript with RNAPII p-CTD.

### CSDE1 regulates the interaction between RNAPII p-CTD and CDK7

**Fig. 7:**
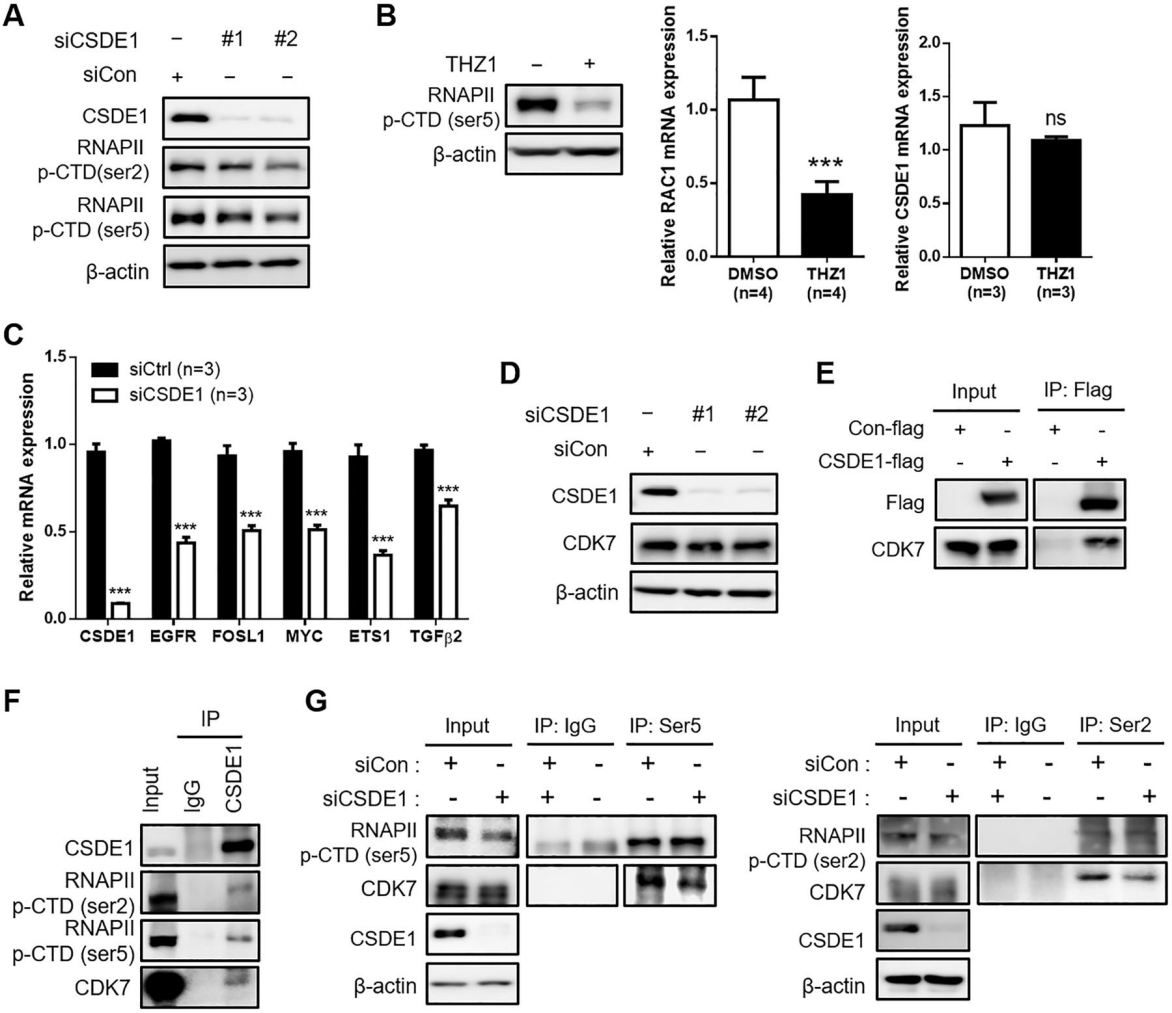
CSDE1 deficiency reduced interaction between RNAPII and CDK7. **A** Western blot analysis showed expression of CSDE1 and RNAPII (ser2, ser5) in *CSDE1* siRNA-treated MDA-MB-231 cells. **B** Western blot analysis showing expression of RNAPII (ser5) in MDA-MB-231 cells under THZ1 (500 nM) treatment (left). qRT-PCR analysis of *Rac1* and *CSDE1* expression in MDA-MB-231 cells under THZ1 treatment (right). Data are presented as mean ± SD with 3 or 4 biological replicates. **C** Relative mRNA expression of *CSDE1*, *EGFR*, *FOSL1*, *MYC*, *ETS1* and *TGFβ2* expression in *CSDE1* siRNA-transfected MDA-MB-231 cells. Data are presented as mean ± SD with 3 biological replicates. **D** Western blot analysis showing expression of CSDE1 and CDK7 in *CSDE1* siRNA-treated MDA-MB-231 cells. **E** Interaction of CSDE1 Flag with CDK7 in HEK293T cells transfected with control or CSDE1-Flag-tagged vector. **F** Interaction of endogenous CSDE1 and RNAPII p-CTD (ser2, ser5) with CDK7 in MDA-MB-231 cells. **G** Comparison of interaction between RNAPII p-CTD and CDK7 in Control or *CSDE1* siRNA-transfected MDA-MB-231 cells. ***p < 0.005; ns not significant.

To determine whether CSDE1 binds to regulate the expression of RNAPII p-CTD, we validated RNAPII p-CTD expression in *CSDE1*-knockdown cells. The expression of RNAPII p-CTD was decreased in *CSDE1* siRNA-treated MDA-MB-231 cells (Fig. 7A). In addition, we used THZ1, a CDK7 inhibitor, to downregulate RNAPII p-CTD expression. Since CDK7 controls gene transcription by phosphorylating RNAPII [30], CDK7 inhibitors are widely used to repress RNAPII p-CTD. Downregulation of RNAPII p-CTD expression after THZ1 treatment did not alter *CSDE1* expression, but reduced *RAC1* expression (Fig. 7B), suggesting that decreased RNAPII p-CTD expression is associated with the inhibition of *Rac1* transcription in TNBC cells. In addition, we found that the expression of THZ1 regulated genes, [31] such as *EGFR*, *FOSL1*, *MYC*, *ETS1*, and *TGFβ2*, was reduced in *CSDE1* siRNA-transfected MDA-MB-231 cells (Fig. 7C), implying that CSDE1 is involved in the transcriptional regulation on a global scale, including the regulation of *RAC1*, via regulation of p-RNAPII.

We next evaluated CDK7 expression in *CSDE1*-knockdown TNBC cells to confirm whether CSDE1 regulates RNAPII p-CTD expression via CDK7 regulation. Our results showed that CSDE1 deficiency did not alter CDK7 expression (Fig. 7D). However, the interaction between CSDE1 and CDK7 was identified (Fig. 7E, F), and CSDE1 was found to form a complex with RNAPII p-CTD and CDK7 (Fig. 7F). Based on this interaction, we assumed that the interaction between CDK7 and RNAPII p-CTD is regulated by CSDE1. To confirm this hypothesis, RNAPII p-CTD was pulled down from the lysates of *CSDE1*-knockdown MDA-MB-231 cells. Interestingly, CDK7 coprecipitated with RNAPII p-CTD in the control MDA-MB-231 cells, whereas less precipitation of CDK7 was observed in *CSDE1*-knockdown MDA-MB-231 cells (Fig. 7G). Therefore, CSDE1 deficiency led to weak interaction between RNAPII p-CTD and CDK7 to reduce RNAPII p-CTD expression, suggesting that CSDE1 regulates *Rac1* transcription by regulating the interaction between RNAPII p-CTD and CDK7 in TNBC cells.

### miR-371b-5p targeted CSDE1/RAC1 axis and regulated TNBC aggressiveness

**Fig. 8:**
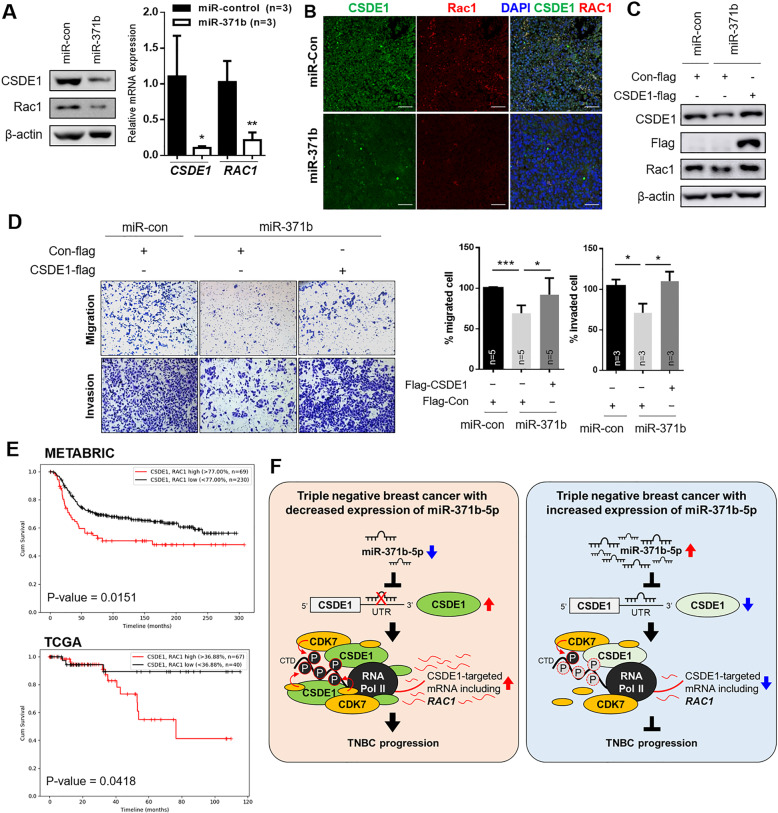
miR-371b-5p modulates CSDE1/Rac1 expression in TNBC cells. **A** Western blot analysis showing expression of both CSDE1 and Rac1 in MDA-MB-231 cells stably expressing NC mimic or miR-371b mimic (left). qRT-PCR analysis of CSDE1 and Rac1 mRNA in MDA-MB-231 cells stably expressing NC mimic or miR-371b mimic (right). Data are presented as mean ± SD with 3 biological replicates. **B** Representative images of CSDE1 and Rac1 fluorescence staining in miR-371b-overexpressing tumors and control xenografts. **C** The expression of Rac1, CSDE1, and Flag was analyzed via western blotting after treatment with Flag-tagged CSDE1 (CSDE1-flag) or control (Con-flag) in MDA-MB-231 cells stably overexpressing miR-con or miR-371b. **D** Representative images of migration and invasion assays in miR-con- and miR-371b-overexpressing cells with CSDE1-flag or Con-flag. The bar graph illustrates quantification of migrated or invaded cells in each group. Data are presented as mean ± SD with 3 or 5 biological replicates. **E** The disease-free survival analysis depending on CSDE1 and Rac1 expression in METABRIC and TCGA subtypes. **F** Schematic illustration of CSDE1-RNAPII(p-CTD)-CDK7/Rac1 axis regulated by miR-371b-5p in TNBC. miR-371b-5p directly regulates the expression of CSDE1 that plays as a connector between RNAPII p-CTD and CDK7. CSDE1 forms complex with RNAPII p-CTD and CDK7 and increases the expression of CSDE1 targeted genes, including *Rac1*, to induce aggressiveness of TNBC. In CSDE1-deficient TNBC cells, weak interaction between RNAPII p-CTD and CDK7 results in decrease in *Rac1* transcription, thereby leading to inhibition of TNBC progression. *p < 0.05, **p < 0.01, ***p < 0.005.

Since miR-371b directly inhibited CSDE1 expression and CSDE1 regulated the transcription of *Rac1* in TNBC, we investigated the role of the miR-371b-5p/CSDE1/RAC1 axis through restoration of miR-371b-5p in TNBC cells. We first examined Rac1 expression after treatment with miR-371b-5p mimics and a negative control. As shown in Supplementary Fig. 5, Rac1 and CSDE1 expression was reduced in miR-371b-5p-overexpressing TNBC cells. Next, we generated stable MDA-MB-231 cell lines using miR-371b-5p (miR-371b) and miR-con and observed the expression of Rac1 and CSDE1. The protein and mRNA expression of both CSDE1 and Rac1 was markedly downregulated in MDA-MB-231/miR-371b-5p cells (Fig. 8A). In addition, miR-371b-overexpressing xenograft tumors showed decreased expression of CSDE1 and Rac1 compared to the miR-con tumor (Fig. 8B). To explore whether miR-371b-5p modulates the CSDE1/RAC1 axis, we evaluated Rac1 expression by re-expressing CSDE1 in miR-371b-5p-overexpressing cell lines. The expression of Rac1 was significantly restored after increasing the expression of CSDE1 in stable miR-371b-5p-expressing cell lines (Fig. 8C). These data support the finding that miR-371b-5p directly regulates CSDE1 expression and alters the expression of Rac1 in TNBC. Consistently, by restoration of CSDE1 expression in miR-371b-5p-overexpressing MDA-MB-231 cells, the migration and invasion abilities were slightly activated compared to that in miR-371b-5p-overexpressing cells (Fig. 8D). Furthermore, patients with TNBC and high expression of CSDE1 and Rac1 showed poor disease-free survival according to the METABRIC and TCGA data (Fig. 8E).These findings suggest that downregulation of miR-371b-5p alters CSDE1/Rac1 expression and promotes invasiveness in TNBC.

## Discussion

Our data provide evidence that downregulation of miR-371b-5p regulates CSDE1/Rac1 expression and leads to TNBC progression. We demonstrated that miR-371b-5p expression was significantly downregulated in TNBC compared to that in luminal A, luminal B, and HER2 subtypes. By using integrative analysis of miRNA profiling and mRNA expression data, CSDE1 was found to be directly regulated by miR-371b-5p expression, which was considerably reduced in TNBC. We also found that CSDE1 acts as a connector of RNAPII and CDK7 to regulate the interaction between RNAPII and CDK7, thereby affecting the transcription of *Rac1*. Therefore, our study identified not only an association between reduced expression of miR-371b-5p and tumor progression in vitro and in vivo but also the role of oncogenic CSDE1 in regulating *Rac1* transcription in TNBC cells (Fig. 8F).

Aberrant expression of miRNAs has been observed and characterized in several cancer types. Depending on the cellular and molecular mechanisms in cancer, miRNAs can act as tumor suppressors or oncogenes [32, 33]. Based on these observations, miRNAs have emerged as promising prognostic biomarkers through understanding the alteration of miRNA expression in various cancers [34]. We observed that miR-371b-5p expression was significantly downregulated in TNBC, and this reduced expression was positively correlated with poor OS in patients with TNBC, even after receiving chemotherapy. In particular, miR-371b-5p was associated with poor OS in patients with TNBC treated with chemotherapy. Metastatic TNBC (mTNBC) patients experience difficulty in chemotherapy because a targeted therapy to treat patients with TNBC is not yet available [35, 36]. The most important factor in predicting cancer progression in mTNBC is the identification of better therapeutic options. Detection for prognosis using miR-371b-5p could have a high propensity for predicting cell invasion and migration in TNBC. Therefore, miR-371b-5p could be a promising prognostic marker in TNBC owing to its tumor-suppressive role.

In addition, we showed that CSDE1 expression is upregulated by reduced expression of miR-371b-5p in TNBC. In the Chinese breast cancer set, *CSDE1* was determined to be one of the principal component genes that predicted high-risk groups using comparative genome hybridization and gene expression microarray [37]. In addition, CSDE1 expression was upregulated and associated with the aggressiveness of melanoma. Indeed, RNA sequencing, ribosomal profiling, and iCLIP sequencing suggested that CSDE1 regulated pro-metastatic factor RNA regulons and mediated invasion and metastasis by regulating translation elongation of vimentin and *Rac1* mRNA in melanoma [20]. CSDE1 was identified as a connector between proteins and RNAs in many ways to reprogram translation [28]. We found that CSDE1 directly regulated RAC1 translation by binding its mRNA transcript with RNAPII and CDK7 in TNBC. CDK7 is involved in transcription initiation and elongation by regulating phosphorylation of RNAPII [29, 31]. Interestingly, it has been reported that CDK7 plays an essential role in regulating the expression of clusters of genes in TNBC [31]; this finding suggests that identification of CDK7-related factors involved in transcriptional regulation is critical for understanding TNBC progression. Here, we identified a novel role of CSDE1 by revealing that CSDE1 regulated the interaction between RNAPII and CDK7 to control *Rac1* transcription in TNBC. The association between RNAPII and CDK7 is well documented, but there are few reports suggesting connectors between these two proteins. We found that downregulation of CSDE1 expression decreased RNAPII p-CTD expression via reduced interaction between RNAPII p-CTD and CDK7 in TNBC cells. These data thus provide a novel mechanism of *Rac1* transcriptional regulation involved in the CSDE1-RNAPII-CDK7 complex.

Our study provides insight into the mechanisms by which reduced miR-371b-5p expression in TNBC promotes aggressive tumor progression in vitro and in vivo. Moreover, inhibition of expression of *CSDE1*, which is a direct target gene of miR-371b-5p, regulates the expression of a pro-metastatic gene, *RAC1*, by controlling the interaction between RNAPII p-CTD and CDK7. Collectively, we revealed the oncogenic function of miR-371b/CSDE1 involved in *Rac1* transcription regulation, thus providing a basis for the pathological mechanism of TNBC along with potential biomarkers for TNBC.

## Materials and methods

Methodology is described in the Supplementary Methods file.

## Supplementary information

Supplementary methods

Supplementary data
