# Supplementary material for: Reduced miR-371b-5p expression drives tumor progression via CSDE1/RAC1 regulation in triple-negative breast cancer
Source: Oncogene. 2022 Apr 30;41(22):3151–61. doi: 10.1038/s41388-022-02326-6 (PMC9135623; doi:10.1038/s41388-022-02326-6)
Supplement: Supplementary file 1 — Supplementary methods [file 41388_2022_2326_MOESM1_ESM.pdf]

Supplementary Methods

Breast cancer patient sample

Patients who were diagnosed with and treated for breast cancer at Seoul National University Hospital were included in this study. Tumor and adjacent normal tissues were surgically resected from patients in each subtype group at Seoul National University Hospital. Ten tumors and 10 paired adjacent normal tissues were used for the miRNA microarray analysis, and the numbers and types of patients were as follows: luminal A (n=2), luminal B (n=2), HER2 (n=3), and TNBC (n=3). Thirteen tumor tissues were analyzed to confirm miR-371b-5p expression using qPCR. The range of initial surgical age of the patients used for the miRNA microarray analysis was 36–59 years. Tumor blocks were harvested from the tumor cores and normal tissues were derived from the same patients at a location at least 5 cm away from the cancer lesion. Absence of cancer cells in the normal tissues was confirmed through H&E staining and microscopic examination. Tissues were stored in nitrogen tanks at a tissue bank (Laboratory of Breast Cancer Biology, Seoul National University College of Medicine, Seoul, Korea).

Ethics approval and consent to participate

Tumor and adjacent normal tissues were surgically resected from patients in each subtype group at Seoul National University Hospital. Informed consent was obtained from all patients before collection of the specimens, and the study was approved by the Institutional Review Board of Seoul National University Hospital (IRB number: 1704-019-843).

miRNA and mRNA profiling

Total RNA was isolated using TRIzol Reagent (Thermo Fisher Scientific, MA, USA) and purified using chloroform and isopropanol for miRNA microarray analysis on an Agilent G4872A platform (Illumina, Inc., CA, USA). First, isolated total RNA was amplified using an Illumina TotalPrep RNA amplification kit (Invitrogen, CA, USA) to yield biotinylated cRNA,

according to the manufacturer's instructions. The labeled cRNA was hybridized to each HumanHT-12 expression v2 BeadChip array. The value of probe signals was transformed into log-scale and normalized using the quantile method. Data analysis was performed as described previously (1).

Survival and molecular subtype association analysis

The Kaplan-Meier (KM) plotter tool was used to show the correlation between miR-371-5p expression or CSDE1 and overall survival (OS) in patients with breast cancer (2). Survival analysis according to CSDE1 and RAC1 expression was analyzed using the Cancer Target Gene Screening Tool (3). Subtype-specific *CSDE1* expression analysis was performed using the cBioportal (4, 5) on The Cancer Genome Atlas (TCGA) breast cancer dataset (6). *CSDE1* mRNA expression was classified using PAM50 subtyping. TNBC patients were automatically split by KMplot into two groups with high (n=52) and low (n=151) levels of miR-371b-5p (expression range: 6-7, cutoff value: 6.51, HR: 0.44). Chemotherapy-treated TNBC patients were automatically split by KMplot into two groups with high (n=31) and low (n=79) levels of miR-371b-5p expression (expression range: 6-7, cutoff value: 6.5, HR: 0.38). The settings for specifying the chemotherapy group were as follows: Cohorts, systemically treated patients; ER-positive endocrine therapy, any; Chemotherapy, include all. Lymph node-positive TNBC patients were automatically split by KMplot into two groups with high (n=21) and low (n=46) levels of CSDE1 expression (expression range: 921-7722, cutoff value: 3758). The settings for specifying the lymph node-positive TNBC patients were as follows: Lymph node status, positive; Intrinsic subtype, basal. The relative expression value was indicated as the number of standard deviations away from the mean expression value in the reference population (Z-score).

Animal studies

Female Balb/c Nude mice at 7 weeks from Orient Bio (Korea) were used in the xenograft studies. All procedures for nude mice were approved by the Animal Care and Use Committee of Yonsei University Medical School and performed in specific pathogen-free facility under conditions in accordance with the Guidelines for the Care and Use of Laboratory Animals of Yonsei University Medical School. All animal experiments were approved by the institutional review board (IRB) of the Yonsei university college of medicine (Approved animal experiment number : 2018-0155). Mice were randomly allocated to different groups (miRNA-control, n = 8; miR-371b-5p, n=7) and were subcutaneously inoculated with 1 × 10^6 stable MDA MB-231-(miRNA control) and MDA-MB-231-(miR-371b-5p) cells into each flank under 150 μl of saline/zoletil/rompun (7:1:1) anesthesia. From palpable tumor formation until termination, tumor sizes were measured every 2–3 days using calipers, and tumor volume was calculated using the following formula: length × width^2 × 0.5236. Mice were sacrificed in a 7.5% CO_2 chamber, and tumors were harvested for immunohistochemical and other analyses.

Cell lines

MCF10A, SK-BR-3, T47D, BT-549, MDA-MB-157, and MDA-MB-468 cells were purchased from ATCC (Manassas, VA, USA). MCF7, ZR-75-1, HCC-1937, MDA-MB-231, and Hs578T cells were purchased and authenticated in the Korean Cell Line Bank (Seoul, Korea). All human breast cancer cells were characterized and authenticated in cell biology collection using short tandem repeat DNA profiles. Human breast cancer cells were grown in DMEM (SK-BR-3, MDA-MB-157, MDA-MB-468, and HEK293T) or RPMI (MCF7, T47D, BT-549, and MDA-MB-231) supplemented with 10% fetal bovine serum (FBS) (HyClone™, GE Healthcare, IL, USA). MCF10A breast cancer cells were maintained in DMEM/F-12 with 25% horse serum (Gibco®, Thermo Fisher Scientific), 20 ng/ml EGF (Peprotech Inc., NJ, USA), 10 μg/ml insulin (Sigma-Aldrich, MO, USA), 0.5 μg/ml hydrocortisone (Sigma-

Aldrich), and 100 ng/ml cholera toxin (Sigma-Aldrich). The cell lines used in this study were grown in a humidified incubator at 37 °C and 5% CO_2.

Generation of stable cell lines

Lentiviruses expressing miR-371b-5p and control miRNA (miR-con) vector were purchased from Applied Biological Materials, Inc. (BC, Canada). For generation of *CSDE1*-knockout cell lines, a sequence targeting *CSDE1* that has no predicted off-targets obtained from CHOPCHOP v2 (7, 8) was inserted into the lentiCRISPR v2 vector (Addgene, MA, USA). Lentivirus particles in the culture medium were harvested and added to cells with polybrene. After puromycin (Sigma-Aldrich) selection, a stable clone was obtained and confirmed through qRT-PCR and western blotting for further experiments.

Transfection

The miR-371b-5p mimic (miRVana™, Invitrogen) was reverse-transfected using the siPORT™ NeoFX™ Transfection Reagent (Invitrogen) according to the manufacturer's instructions. The control cells were transfected with miRVana™ miR-negative control mimic (Negative Control #1, Invitrogen). siRNA targeting *CSDE1* (Silencer siRNA, Invitrogen) and siRNA negative control (siRNA Negative Control #1, Invitrogen) were transfected using the Lipofectamine RNAiMAX Transfection Reagent (Invitrogen). Both the CSDE1 (NM_007158) vector (ORF size, 2301bp; RC207101, Origene®, USA) and Empty vector (Origene®) were transfected using the FuGENE® HD Transfection Reagent (Promega, USA), following the manufacturer's instructions.

Immunoprecipitation

Cells were lysed using lysis buffer (50 Mm Tris-Cl, pH 7.5; 150 mM NaCl; 1 mM EDTA; 1% Triton X-100; 0.5% NP-40, and protease inhibitor) and incubated on ice for 5 min. Lysates were clarified by centrifugation for 15 min at 13,000 rpm at 4 °C. For immunoprecipitation, the supernatant was incubated with the primary antibody (Flag, F1804,

Sigma; CSDE1, NBP1-71915, Novus; RNAPII p-CTD Ser2, 61083, Active Motif; RNAPII p-CTD Ser5, ab5408, Abcam) at 4 °C overnight. Magnetic protein G beads (Invitrogen) were added to each sample and incubated at room temperature for 2h. Samples were pulled down and washed three times with wash buffer (50 mM Tris-Cl, pH 7.5; 150 mM NaCl; 1 mM EDTA; 1% Triton X-100, and 0.5% NP-40). The samples were then boiled and loaded for further analysis.

RNA immunoprecipitation (RIP) assay

RIP experiments were performed according to protocol of a previous study (9). Briefly, cells were cross-linked using 1% formaldehyde, followed by addition of 0.2 M glycin. Each sample was centrifuged for 5 min at 4 °C. Cells were resuspended in ice-cold FA lysis buffer containing RNase inhibitor (Promega). The samples were then sonicated and centrifuged. Pre-cleared supernatant was incubated overnight with the primary antibodies under rotation at 4 °C. Anti-CSDE1 (NBP1-71915, Novus), anti-RNAPII Ser2 (ab238146, Abcam), anti-RNAPII ser5 (ab5408, Abcam), anti-normal IgG (12-371, Merck, USA), and anti-Flag (F1804, Sigma) were incubated with protein A/G magnetic beads (Invitrogen) and stirred for 90 min at 4 °C. Pellet beads were washed with ice-cold FA500, LiCl, and TE buffers and eluted with RIP elution buffer containing RNase inhibitor for 10 min at 37 °C. Total RNA was then extracted using TRIzol® (Thermo Fisher Scientific, USA) and phenol:chloroform and reverse transcribed for further qRT-PCR analysis.

RNA extraction and qRT-PCR

Total RNA (500 ng) was isolated using the miRNAeasy kit (Qiagen, Germany) according to the manufacturer's instructions and reverse transcribed using the TaqMan® MicroRNA Reverse Transcription Kit (Applied Biosystems, Waltham, MA, USA) to detect miR-371b-5p expression. Quantitative RT-PCR (qRT-PCR) for miRNA analysis was performed with the TaqMan® Universal PCR Master Mix (Applied Biosystems) using the

LightCycler® System (Roche, Switzerland); RNU48 was used as an endogenous control. The mRNAs for qRT-PCR were extracted using the NucleoSpin® RNA/Protein kit (MACHEREY-NAGEL, Germany). Total RNA was reverse transcribed, and each target gene was detected using specific primers.

Western blotting

Western blotting was performed as previously described (10). Briefly, proteins were isolated using the NucleoSpin® RNA/Protein kit (MACHEREY-NAGEL). Proteins were separated on SDS-PAGE, transferred to PVDF membrane and subjected to immunoblotting. Primary antibodies used in this study were CSDE1 (1:1000, rabbit, ab96124, Abcam, UK), Rac1 (1:1000, mouse, ab33186, Abcam, UK), RNA pol II p-CTD (ser2) (1:1000, rat, 61083, Active Motif, USA; 1:1000, rabbit, ab238146, Abcam), RNA pol II p-CTD (ser5) (1:1000, mouse, ab5408, Abcam, UK), CDK7 (1:1000, mouse, #2916, Cell Signaling, USA), Flag (1:5000, rabbit, F7425, Sigma-Aldrich, USA), and β-actin (1:10000, rabbit, A300-491A, Bethyl Laboratories, USA). β-actin was used as a loading control. Immunoreactive proteins were detected by horseradish peroxidase-conjugated secondary antibodies (1:5000, ADI-SAB-100-J, ADI-SAB-300-J, Enzo Life Sciences, NY, USA) and the enhanced chemiluminescence reagent, EzWestLumi plus (ATTO, JAPAN), and analyzed by Amersham™ Imager 600 (GE healthcare, UK).

Cell viability assay and Caspase-3 activation assay

Cell viability was measured by the CCK-8 assay. Cells were transfected with miRNA or siRNA on a 24-well plate per group at a density. Following cell attachment, CCK-8 labeling mixture (Dojindo Molecular Technologies, Inc., Japan) was added to a single well for each group and incubated for 30 min at 37 °C in the dark. The absorbance at 450 nm was measured using a multi-well spectrophotometer (Synergy HTX; BioTek, VT, USA). Cells were harvested,

and caspase-3 activity was measured using ApoAlert Caspase Colorimetric kit (Clonetech Laboratories, USA) according to the manufacturer's instructions.

Cell cycle analysis

Cells were transfected with miR-negative control mimic or miR-371b-5p mimic. After 48 h, cells were washed, harvested with PBS, and fixed with 70% ethanol at 4 °C overnight. The fixed cells were then centrifuged and washed with PBS, resuspended with PI/RNase staining buffer (BD Biosciences, San Jose, CA, USA), and incubated at room temperature in the dark for 30 min. The DNA content was analyzed using a FACSCanto II flow cytometer (BD Biosciences).

Wound healing assay

The miRNA-transfected cells were incubated for 24 h in 6-well plates until they reached 100% confluence. After overnight incubation in complete medium, cells were scratched using a 200 μl pipette tip, washed with 0% FBS medium, and further incubated in 0% FBS medium. The wound healing area of each sample was calculated using the ImageJ software.

Transwell cell migration and invasion assay

The cells (5×10^4 cells/well) were seeded in upper chambers of the Transwell cell culture insert (Corning, NY, USA) and Matrigel Invasion Chambers (BD Biosciences) for the migration and invasion assays, respectively. The lower chambers contained culture medium supplemented with 10% FBS. After 24–48 h, non-migrated cells or invaded cells in upper chambers were removed using cotton swabs. The membranes were fixed and stained with 0.01% crystal violet (Sigma-Aldrich). The migrated or invaded cells were counted using the i-Solution software (IMT, Gyungnam, Korea) or multi-well spectrophotometer (Synergy HTX; BioTek, VT, USA).

Dual luciferase assay

Human *CSDE1* 3'UTR, which included the predicted miR-371b-5p seed sequence and mutated seed sequences, was inserted into the psiCHECK™-2 vector (Promega, Madison, WI, USA) using the In-fusion® HD Cloning Kit (Clontech Laboratories, CA, USA). This luciferase reporter construct was transfected into HEK293T cells with 15–30 nM solution of miR-371b-5p mimics or negative control miRNA using Lipofectamine 2000 (Invitrogen) according to the manufacturer's instructions. After 48 h, all experimental cells were passively lysed, and reporter activity was measured using the Dual Luciferase Assay System (Promega). The following sizes and positions of the 3'UTR were used in this study: CSDE1 (NM_001007553; 1st seed position, 1122-1128 of CSDE1 3'UTR; 2nd seed position, 1242-1248 of CSDE1 3'UTR), CDC7 (NM_003503; 1st seed position, 1172-1178 of CDC7 3'UTR; 2nd seed position, 822-828 of CDC7 3'UTR), E2F2 (NM_004091; 1st seed position, 1612-1619 of E2F2 3'UTR; 2nd seed position, 2362-2368 of E2F2 3'UTR), MCM4 (NM_005914; 1st seed position, 738-744 of MCM4 3'UTR; 2nd seed position, 1000-1006 of MCM4 3'UTR), NEK2 (NM_002497; 1st seed position, 432-428 of NEK2 3'UTR), PAG1 (NM_018440; 1st seed position, 2519 - 2525 of E2F2 3'UTR; 2nd seed position, 6209 -6216 of E2F2 3'UTR).

Immunohistochemistry and immunofluorescence analyses

For immunohistochemistry analysis, paraffin-embedded slides were probed with the primary antibodies against Ki67 (1:200, rabbit, ab16667, Abcam, UK). Staining was visualized using NovaRED substrate (Vector Laboratories, Cambridgeshire, UK), followed by counterstaining with hematoxylin. For immunofluorescence analysis of paraffin-embedded slides, the primary antibodies against CSDE1 (1μg/ml, rabbit, NBP2-38665, Novus; 1:500, rabbit, ab96124, Abcam, UK) and RAC1 (1:100, mouse, ab33186, Abcam, UK) were incubated with slides overnight at 4 °C. For immunofluorescence staining, the cells were fixed with 10% neutral buffered formalin (BioSesang, Korea) and permeabilized with 0.2% Triton X-100 containing 5% BSA. Thereafter, the cells were incubated overnight with the primary antibodies

(rhodamine-labeled phalloidin, 1:500, R415, Invitrogen; anti-CSDE1, 1:250, rabbit, ab96124, Abcam, UK; anti-Ki67, 1:250, rabbit, ab16667, Abcam, UK; DAPI, 1:1000, Invitrogen, MA, USA) at 4 °C. Fluorescent secondary antibodies (1:1000; A11032, A11034, and A11037, Invitrogen, MA, USA) were used to detect the primary antibodies. Immunofluorescence images were captured at a wavelength of 488 nm or 594 nm, using a confocal laser scanning microscope (LSM-700, Carl Zeiss).

Availability of data and materials

The data that support the findings of this study are available from GSE154255, but restrictions apply to the availability of these data, which were used under license for the current study and therefore are not publicly available. Data are, however, available from the authors upon reasonable request and with permission of the corresponding author.

Statistical analysis

Statistical analyses were performed using the GraphPad Prism 5 software. Data are presented as the mean ± standard deviation (SD). All sample sizes were indicated in the corresponding figure. At least three biological or technical replicates were used for statistical analysis of *in vitro* experiments. For statistical analysis of *in vivo* data, at least four values (n=4) per group were used. The data were analyzed using a two-tailed unpaired student's t-test for comparison between different groups. Statistical significance was set at p <0.05 (*$p < 0.05$, **$p < 0.01$, ***$p < 0.001$).

References

1. Son D, Kim Y, Lim S, Kang HG, Kim DH, Park JW, et al. miR-374a-5p promotes tumor progression by targeting ARRB1 in triple negative breast cancer. Cancer Lett. 2019;454:224-33.
2. Lanczky A, Nagy A, Bottai G, Munkacsy G, Szabo A, Santarpia L, et al. miRpower: a web-tool to validate survival-associated miRNAs utilizing expression data from 2178 breast cancer patients. Breast cancer research and treatment. 2016;160(3):439-46.
3. Kim HY, Choi HJ, Lee JY, Kong G. Cancer Target Gene Screening: a web application for breast cancer target gene screening using multi-omics data analysis. Brief Bioinform. 2020;21(2):663-75.
4. Gao J, Aksoy BA, Dogrusoz U, Dresdner G, Gross B, Sumer SO, et al. Integrative analysis of complex cancer genomics and clinical profiles using the cBioPortal. Science signaling. 2013;6(269):pl1.
5. Cerami E, Gao J, Dogrusoz U, Gross BE, Sumer SO, Aksoy BA, et al. The cBio cancer genomics portal: an open platform for exploring multidimensional cancer genomics data. Cancer discovery. 2012;2(5):401-4.
6. Ciriello G, Gatza ML, Beck AH, Wilkerson MD, Rhie SK, Pastore A, et al. Comprehensive Molecular Portraits of Invasive Lobular Breast Cancer. Cell. 2015;163(2):506-19.
7. Labun K, Montague TG, Gagnon JA, Thyme SB, Valen E. CHOPCHOP v2: a web tool for the next generation of CRISPR genome engineering. Nucleic acids research. 2016;44(W1):W272-6.
8. Montague TG, Cruz JM, Gagnon JA, Church GM, Valen E. CHOPCHOP: a CRISPR/Cas9 and TALEN web tool for genome editing. Nucleic acids research. 2014;42(Web Server issue):W401-7.
9. Selth LA, Gilbert C, Svejstrup JQ. RNA immunoprecipitation to determine RNA-protein associations in vivo. Cold Spring Harbor protocols. 2009;2009(6):pdb prot5234.
10. Park JW, Kim Y, Lee SB, Oh CW, Lee EJ, Ko JY, et al. Autophagy inhibits cancer stemness in triple-negative breast cancer via miR-181a-mediated regulation of ATG5 and/or ATG2B. Mol Oncol. 2022.
