# Supplementary material for: Reduced miR-371b-5p expression drives tumor progression via CSDE1/RAC1 regulation in triple-negative breast cancer
Source: Oncogene. 2022 Apr 30;41(22):3151–61. doi: 10.1038/s41388-022-02326-6 (PMC9135623; doi:10.1038/s41388-022-02326-6)
Supplement: Supplementary file 2 — Supplementary data [file 41388_2022_2326_MOESM2_ESM.pdf]

Supplementary Data

Supplementary Figure 1.

A

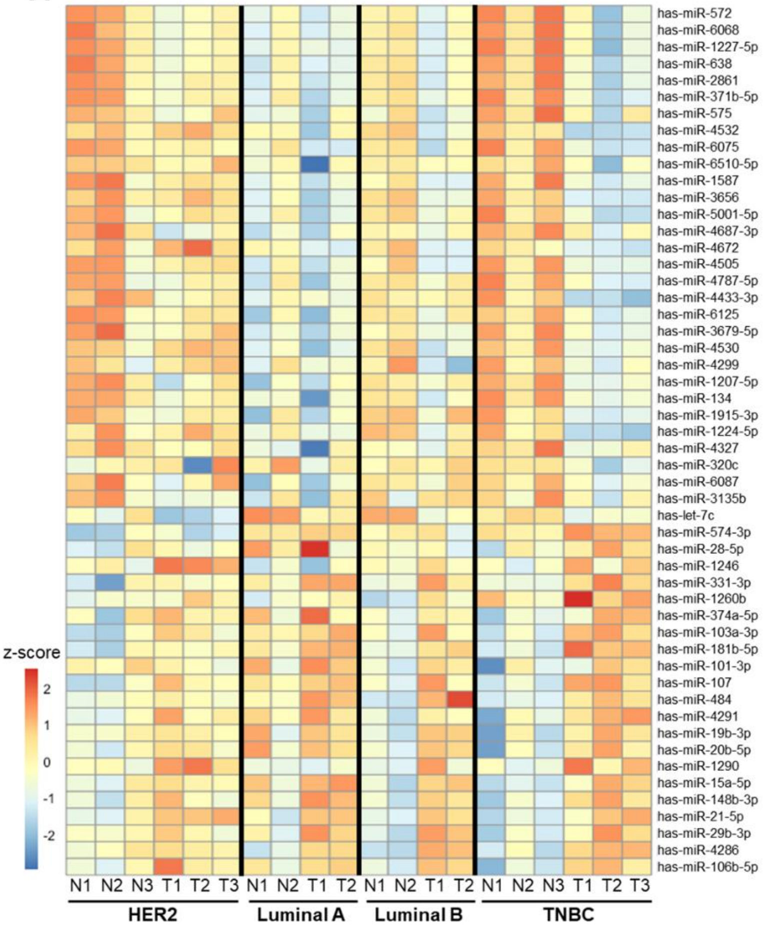

B

miRNAs	FC	p-value
hsa-miR-572	-11.817218	0.01742299
hsa-miR-6068	-10.557656	0.01877951
hsa-miR-1227-5p	-10.273291	0.01178495
hsa-miR-638	-6.798754	0.01005961
hsa-miR-2861	-6.178	0.01745894
hsa-miR-371b-5p	-5.98811	0.01301863
hsa-miR-575	-5.454286	0.01800047
hsa-miR-4532	-5.40086	0.01990115
hsa-miR-6075	-4.656335	0.02309433
hsa-miR-6510-5p	-4.64697	0.04830983
hsa-miR-1587	-4.461357	0.03572365
hsa-miR-3656	-4.210376	0.02029121
hsa-miR-5001-5p	-3.799419	0.01224444
hsa-miR-4787-5p	-3.107446	0.03853066
hsa-miR-4433-3p	-3.085315	0.04410068
hsa-miR-6125	-3.00856	0.03107027
hsa-miR-3679-5p	-2.929525	0.03744213
hsa-miR-1915-3p	-2.445356	0.04535425
hsa-miR-28-5p	2.275865	0.03280291
hsa-miR-374a-5p	3.438045	0.01298822
hsa-miR-103a-3p	3.929402	0.0121531
hsa-miR-181b-5p	4.025692	0.03546355
hsa-miR-107	4.1741	0.0167299
hsa-miR-4291	4.256226	0.04264246
hsa-miR-20b-5p	5.545285	0.04361374
hsa-miR-148b-3p	7.41267	0.02329405
hsa-miR-106b-5p	14.105772	0.03557312

Supplementary Figure 1. Analysis of miRNA microarray data of breast cancer tissues

(A) A heatmap showing the 52 miRNAs in matched normal tissues and tumors in four breast cancer subtypes. Z-score (normalized expression levels) are represented by colored bars; higher (red) or lower (blue). (B) Twenty-seven miRNAs that is differentially expressed in patients with TNBC compared to other subtypes (Luminal A, Luminal B, and HER2).

Supplementary Figure 2.

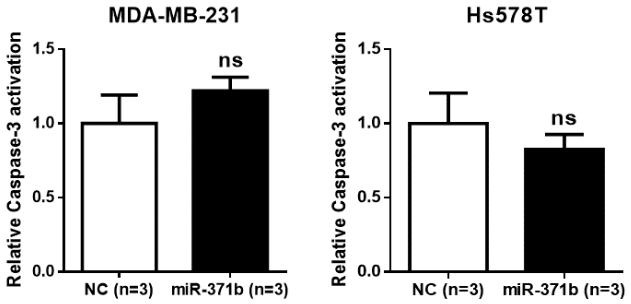

Supplementary Figure 2. Effect of miR371b-5p on apoptosis of MDA-MB-231 and Hs578T

Relative Caspase-3 activation in MDA-MB-231 and Hs578T cells after treatment with miR-371b-5p mimics or negative control (NC) mimic. Data are presented as mean ± SD with 3 biological replicates. ns, not significant.

Supplementary Figure 3.

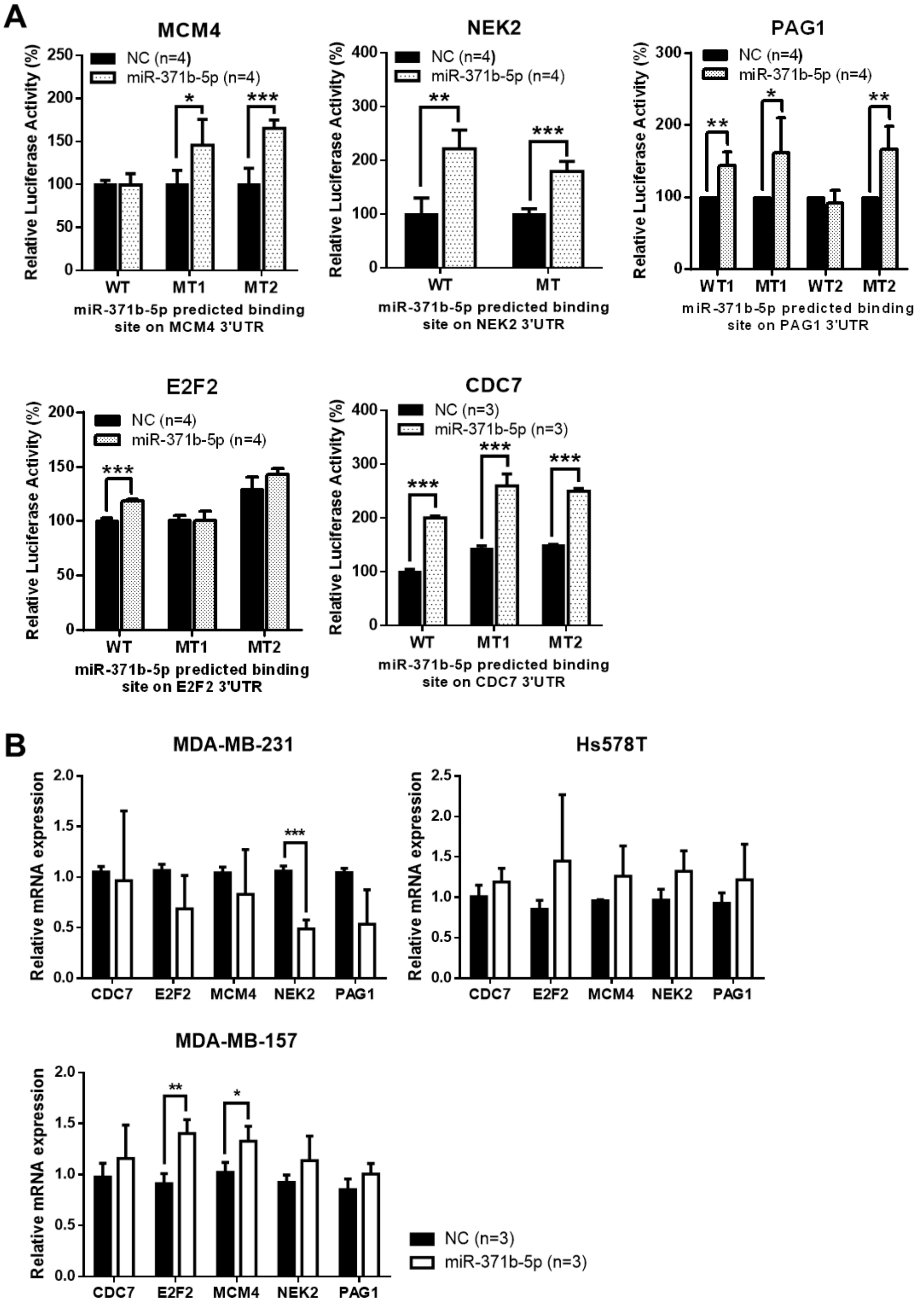

Supplementary Figure 3. Luciferase activity of each gene with miR-371b-5p

(A) Relative Luciferase activity was analyzed for validation as miR-371b-5p target gene. WT (wild type) plasmid contains potential miR-371b-5p binding site, but MT (mutant type) has

mutant binding site. NC, negative control mimic; miR-371b-5p, miR-371b-5p mimic. Data are presented as mean ± SD with 3 or 4 biological replicates. (B) Relative mRNA expression of CDC7, E2F2, MCM4, NEK2, and PAG1 in miR-371b-5p overexpressed MDA-MB-231, MDA-MB-157, and Hs578T cells. Data are presented as mean ± SD with 3 biological replicates. *$p < 0.05$, **$p < 0.01$, ***$p < 0.005$.

Supplementary Figure 4.

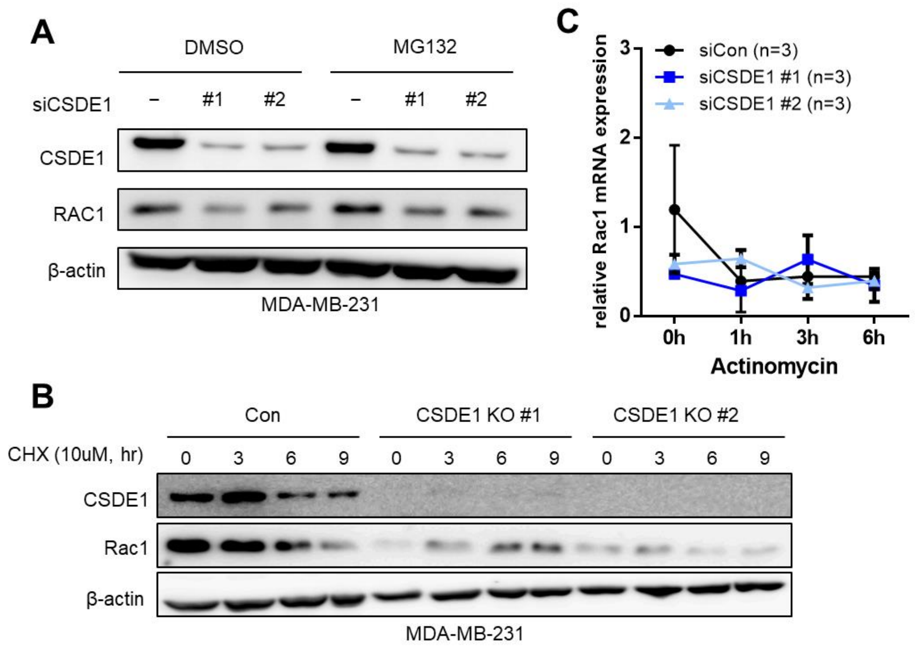

Supplementary Figure 4. Effect of downregulation of CSDE1 on RAC1 protein and mRNA stability

(A) Western blot analysis of CSDE1 and Rac1 expression in MDA-MB-231 transfected with siControl or siCSDE1 under MG132 (proteasome inhibitor) treatment. (B) Western blot analysis of CSDE1 and Rac1 expression in each stably knock-out CSDE1 and control cells in MDA-MB-231 under cycloheximide (inhibitor of protein biosynthesis) treatment. (C) Relative Rac1 mRNA expression in MDA-MB-231 transfected with siControl or siCSDE1 under actinomycin treatment at indicated time points. Data are presented as mean ± SD with 3 biological replicates.

Supplementary Figure 5.

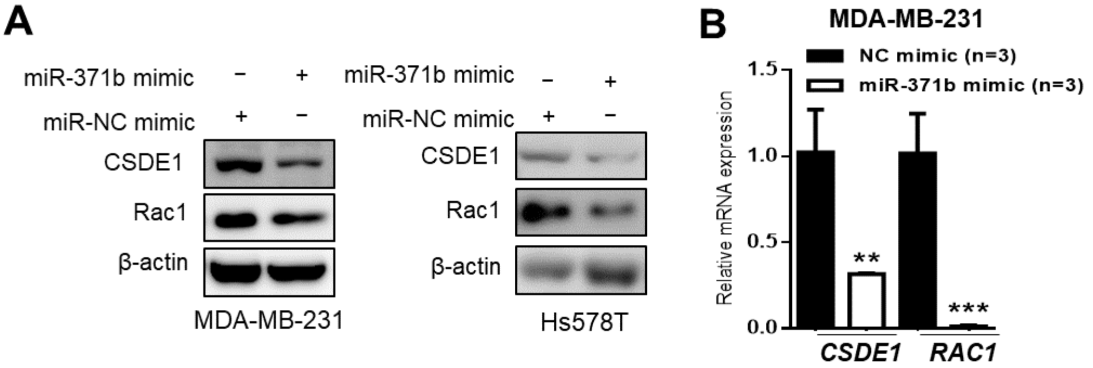

Supplementary Figure 5. Ectopic expression of miR371b mimic decreases CSDE1 and Rac1 expression

(A) Western blot analysis showed expression of CSDE1 and Rac1 in MDA-MB-231 and Hs578T cells incubated with miR-371b mimics or negative control. (B) The qRT-PCR analysis of CSDE1 and Rac1 mRNA in MDA-MB-231 with NC mimic or miR-371b mimic. Data are presented as mean ± SD with 3 biological replicates. **p<0.01, ***p<0.005.
